# *“Once you open that door, it’s a floodgate*”: Exploring work-related grief among community service workers providing care for structurally vulnerable populations at the end of life through participatory action research

**DOI:** 10.1177/02692163221139727

**Published:** 2022-12-02

**Authors:** Melissa Giesbrecht, Ashley Mollison, Kara Whitlock, Kelli I Stajduhar

**Affiliations:** 1 Institute on Aging and Lifelong Health, University of Victoria, Victoria, BC, Canada; 2 School of Nursing, University of Victoria, Victoria, BC, Canada

**Keywords:** Health equity, community-based participatory action, end-of-life care, grief, emotional distress, vulnerable populations, homeless persons, poverty

## Abstract

**Background:**

At the end of life, people experiencing structural vulnerability (e.g. homelessness, poverty, stigmatization) rely on community service workers to fill gaps in access to traditional palliative services. Although high levels of burnout are reported, little is known about these workers’ experiences of grief.

**Aim:**

To explore community service workers’ experiences of grief to identify ways of providing more tailored, meaningful, and equitable supports.

**Design:**

A community-based participatory action research methodology, informed by equity perspectives, was employed.

**Setting/participants:**

In an urban center in western Canada, community service worker (primary) participants (*n* = 18) were engaged as members of an action team. A series of 18 action cycles took place, with secondary participants (*n* = 48) (e.g. palliative, social care, housing support, etc.) being recruited throughout the research process. Focus groups (*n* = 5) and evaluative interviews (*n* = 13) with participants were conducted. Structured observational field notes (*n* = 34) were collected during all team meetings and community interventions. Interpretive thematic analysis ensued through a collaborative and iterative process.

**Results:**

During initial meetings, action team participants described experiences of compounding distress, grief, and multiple loss. Analysis showed workers are: (1) grieving as family, not just providers; (2) experiencing complex layers of compounded grief; and (3) are fearful to open the “floodgates” to grief.

**Conclusions:**

Findings contribute to our understanding on the inequitable distribution of grief across society. A collective and material response is needed, including witnessing, acknowledging and valuing the grief process; facilitating community wellness, collective grieving, and advocacy; and providing training and tools in a palliative approach to care.

**What is already known?**

- Grief and bereavement literature tends to emphasize the “individual” experience, leaving the broader social contexts underexamined.
- Populations experiencing structural vulnerability face significant gaps in access to care, resulting in community service workers stepping up to fill this void, including at the end-of-life.
- Although little research exists, some studies indicate that witnessing numerous unjust and preventable deaths results in these workers experiencing levels of grief and distress similar to emergency responders.

**What this paper adds?**

- Highlights the critical role community service workers play in the provision of care for structurally vulnerable populations at the end-of-life.
- Provides details on how these workers may experience grief as “de facto” family members, in complex and compounded ways, while having to navigate between personal/professional roles with little workplace recognition, guidance, tools, or support.
- Contributes to our understanding on the inequitable distribution of grief in our society by demonstrating how structurally vulnerable populations, and those who provide their care through life and death, suffer from a disproportionate level of collective grief.

**Implications for programs/supports**

- A collective and material response is required, which includes witnessing, acknowledging, and valuing the grief of community service workers and the lives they are grieving.
- Rather than pathologizing personal experiences of grief, interventions and supports may need to take an alternative “side-door” approach, which focuses on facilitating collective care/grieving, community wellness, and community advocacy.
- Workplace supports oriented toward a palliative approach to care may help to recognize and sustain workers in this valuable caregiving role.

## Background

Death is a universally experienced life event; however, grief, loss, and bereavement are not experienced equitably across populations.^1–6^ Grief, which encompasses bereavement and other experiences of loss,^7^ is often understood as an individual experience,^5^ leaving the broader social contexts within which death and dying occur underexamined.^5^ Relations of power, social processes, and intersecting social factors (e.g. economic status, culture, gender, race, etc.) coalesce to create patterned experiences of grief and distress at the community and population level.^7–10^ Viewed through an equity lens, research demonstrates that the disproportionate burden of disease and negative health outcomes among populations experiencing deficits in the social determinants of health (e.g. income, food security, housing, etc.)^11^ leads to an excessive number of preventable, early, and/or undignified deaths,^12^ which in turn can result in a disproportionate amount of distress, grief, suffering, and trauma within those communities.

“Structural vulnerability” is a concept that encapsulates how people or populations located in the lower rankings of our social hierarchy experience constrained choices and opportunities, as well as amplified risk, harm, and negative health outcomes.^13–15^ Social hierarchies are produced by social and structural inequities, which are the manifestations of various relations and effects of power, patterns of privilege and oppression, and built-in structures of government, institutions, or social networks that reify extreme social disadvantage (e.g. homelessness, poverty, etc.).^13,15,16^ In this study, structurally vulnerable populations are defined as people experiencing extreme poverty and forms of homelessness, alongside various facets of racism, trauma, social exclusion, stigmatization, mental health issues, substance use, criminalization, and/or disabilities. At the end of life, structural vulnerabilities often become amplified, resulting in deaths marked by limited access to supports, inadequate pain management and suffering, and occurring in less-than-ideal places.^13,17^

Evidence suggests that gaps in access to traditional palliative services and social support systems that structurally vulnerable populations experience results in considerable voids, which often are filled by friends, street family, and/or support from community service workers.^4,17–19^ For this analysis, we define these workers as including those who are employed as outreach, support, peer, housing/shelter workers and case managers. Although community service workers are not traditionally conceived of as a palliative care support, research, for example by Wright et al.,^18^ suggests that they are going beyond formal employment roles to fill system gaps for their clients. This “under-the-radar” caregiving is resulting in significant levels of stress, role strain, and a “shadow epidemic” of compounding grief and distress, impacting their capacity to provide needed palliative care.^18,20–23^ Although little research focusses on the end-of-life context, evidence indicates that witnessing numerous unjust and preventable deaths (e.g. overdose response setting) results in workers experiencing levels of grief and distress similar to emergency responders.^3,4,18,20,23^ Considering these workers’ critical role in the provision of community-based palliative care for structurally vulnerable populations, there is a need to gain a better understanding of their work-related grief and distress in order to identify how best to support them in this often-invisible and undervalued care work.

### Aim

The catalyst for the current study stems from findings of our team’s previous ethnographic study that aimed to explore access to care for structurally vulnerable populations at the end of life in an urban region in western Canada.^4,13,17^ Specifically, our previous research identified a lack of awareness surrounding palliative approaches to care as well as tools to support community service workers in identifying and caring for people in need. From here, our current community-based participatory action research study developed, with the aim to integrate a palliative approach to care into the work of community service workers who already are providing care to structurally vulnerable populations.

During the research process, significant emotional, physical, and social impacts from work-related grief and distress emerged, leading to the current analysis, which aims to examine community service worker participants’ experiences of grief and distress in order to identify how best to provide more tailored, meaningful, and equitable supports.

## Methods

### Methodology

A community-based participatory action research methodology,^24^ informed by equity-perspectives,^25^ was employed. Community-based participatory action is an approach to research that involves researchers and community stakeholders as partners in all steps of the research process with the goals of educating, improving practice, and/or bringing about social change.^26^ While participatory action was the overarching methodology of our study, specific “action cycles” involving ongoing analysis, dialog, and interventions were also informed by planned-action theories^27^ and knowledge-as-action frameworks.^28^

### Setting

The research took place in an urban center in western Canada from July 2018 to December 2019.

### Recruitment, participants, and the participatory action research process

**Figure 1.**
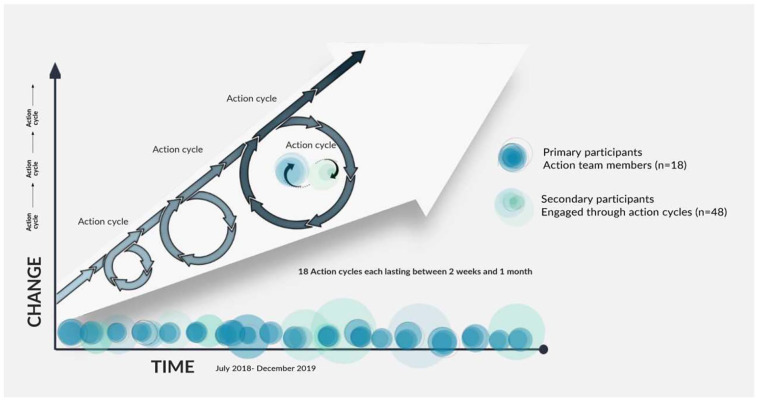
Participatory action research process.

**Figure 2.**
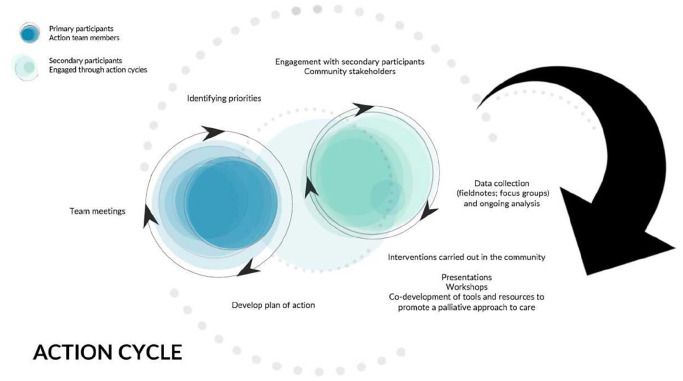
Action cycle process.

Our research process began with development of the Action team, which was to be comprised of academic team members and local community service workers who were identified as playing critical roles in the provision of care for structurally vulnerable populations at the end of life. Community service workers who were previously engaged in our original study were recruited for the current study, thus building upon existing relationships, via written and verbal invitations. Upon consenting to participate, our first action team meeting took place to co-define the aim of the current study, which was to focus on identifying factors contributing to the lack of palliative approaches to care provision for structurally vulnerable populations. From here, a series of 18 action cycles took place (Figure 1), which involved various elements, including engagement with secondary participants identified during action cycles as key stakeholders for successful community interventions (Figure 2). Secondary participants were identified and recruited throughout the study via written and verbal invitations.

### Participant engagement and data collection

Multiple team meetings occurred during each action cycle. Meetings generally lasted 2 h and were attended by our primary participants regularly, with secondary participants joining when relevant to the “action/concern” being addressed. Primary and secondary participants sought permission from their organizations to participate during paid working hours. In some cases, researchers provided additional information to, and sought permission from, organizations on behalf of workers. For organizations operating on a stipend basis, a $25/h stipend ($50 for 2 h) was provided directly to the participant. Each team meeting concluded with participants providing evaluative feedback on any learnings, emerging ideas for practice, and remaining questions/concerns. This feedback informed proceeding meeting agendas, community interventions, and ultimately, the direction of our research study.

Demographic information was collected from all primary (*n* = 18) and secondary participants (*n* = 48). We also conducted focus groups (*n* = 5) with primary and secondary participants and evaluative interviews (*n* = 13) with primary participants at the end of the study. Structured observational field notes (*n* = 34) were collected during every team meeting and community intervention.

### Ethics

All participants provided written and ongoing verbal, informed consent and ethics was approved from the University of Victoria Human Research Ethics Board (18-002) and the Health Research Ethics Board of Island Health (J2018-013).

### Analysis

Analysis was iterative and ongoing throughout the research process, with issues of concern/priority (i.e. themes) emerging from participants, which were then reflected upon and co-analyzed during team meetings. An interpretive thematic^29^ process was used to interactively engage primary participants to reflect on and confirm emerging findings. Analysis involved fieldnotes and focus group/interview transcripts being imported into NVivo™. Three academic team members reviewed all data and, informed by primary participant interpretations, reached consensus regarding the identified themes found. A coding scheme was developed using a hybrid process of deductive and inductive coding, which aimed to capture and organize data according to themes that were both emergent and descriptive of the research process.^30^ To enhance consistency and rigor, one team member conducted all data coding according to the thematic scheme created. A final 2-day action team meeting/retreat was held to interrogate and confirm the findings. A key finding pertaining to the significant levels of grief and distress that workers were experiencing emerged. A refined thematic scheme was then developed and applied to the “grief” data, with these specific findings confirmed by primary participants for accuracy.

### Results

Primary participants comprising our action team (*n* = 18) represented six unique local housing and health support organizations and the health authority (see Table 1). Secondary participants engaged through our action cycles (*n* = 48) represented various local health, palliative, social care, and housing organizations (see Table 2).

**Table 1.** Primary participants – action team members (*n* = 18).

Characteristics	Number of participants
Gender	
Female	13
Male	5
Age	
20–29 years	4
30–39 years	3
40–49 years	5
50–59 years	3
60+ years	2
Did not respond	1
Highest level of education	
Post graduate degree	4
University degree	6
Attended university	1
College diploma	5
High school	1
Did not respond	1
Role	
Outreach worker	5
Case manager	4
Housing/shelter worker	4
Nurse	2
Counselor/social worker	2
Physician	1
Place of employment	
Community health and support services	9
Community housing/shelter	5
Health service organization	4
Length of time in current role	
Less than 1 year	3
1 year to 4 years	9
5 years–9 years	2
10+ years	2
Did not respond	2
Received formal palliative care training	
Yes	5
No	12
Did not respond	1
Received formal training to work with people who experience mental health issues	
Yes	14
No	3
Did not respond	1
Received formal training to work with people who use substances	
Yes	10
No	4
Did not respond	4

**Table 2.** Secondary participants – engaged through action cycles (*n* = 48).

Characteristics	Number of participants
Gender	
Female	31
Male	15
Did not respond	2
Age	
20–29 years	3
30–39 years	11
40–49 years	7
50–59 years	10
60+ years	7
Did not respond	10
Highest level of education	
Post graduate degree	13
University degree	12
Attended university	5
College diploma	5
High school	3
Some high school	3
Did not respond	7
Role	
Counselor/social worker	18
Community service worker	13
Manager/coordinator	7
Health professional	5
Peer advisory volunteer	5
Place of employment	
Health or palliative care organization	26
Community health and support service	12
Community housing/shelter	10
Length of time in current role	
Less than 1 year	8
1 year to 4 years	16
5 years–9 years	5
10+ years	8
Did not respond	11
Received formal palliative care training	
Yes	13
No	26
Did not respond	9
Received formal training to work with people who experience mental health issues	
Yes	24
No	15
Did not respond	9
Received formal training to work with people who use substances	
Yes	25
No	14
Did not respond	9

Initial action team meetings focused on discussing the provision of care to clients and peers at the end of life. However, this topic “*opened the floodgates*” for primary participants, resulting in intensely emotional sessions, with described experiences of profound grief and distress. In these meetings, grief was not only expressed with emotions of sadness, but also anger, frustration, and despair. These emotions emerged because our research process provided participants with a safe space to recognize, express, and process these experiences collectively, demonstrating how the original study objectives became a “side door” into an informal grief and bereavement support process. Consequently, meeting agendas shifted toward first acknowledging and valuing this collective grief and creating a safe space for participants to process these emotions before moving forward with addressing the original study objectives. Interpretive thematic analysis of what participants openly shared and articulated in transcript and fieldnote data revealed three findings, specifically that workers are: (1) grieving as family, not just providers; (2) experiencing complex layers of compounded grief; and (3) are fearful of opening the “floodgates” with no space or time to grieve.

### “It breaks my heart. . .”: Grieving as family, not just providers

Participants explained how a substantial portion of the people they support are not in regular contact with families of origin, while many of their friends are experiencing structural vulnerabilities themselves and therefore not always able or available for the immense effort required in caregiving. At the same time, to provide quality care and support to their clients and peers, workers described being the “constant” person in the lives of people they support and emphasized the importance of building deep, trusting relationships. These resulting close emotional connections were described by participants as often evolving into “de facto” familial relationships, which also meant that clients looked to them in times of illness, need, and/or crisis. As one primary participant explains during an interview:

“*You become family for people, like and I do very deeply feel that. . . almost no one that I work with really has contact with their biological families and the relationships that they have with their peers are often, like, tenuous and complex and I’m a person in their life that is only there for them.”*

Participants commonly stated how they truly “*love*” their clients, as this primary participant elaborated, ***“*** *not just as people in my life, like ‘I love my clients’, but just love them as human beings.”* Therefore, when a client is dying, workers described experiencing a tremendous amount of anticipatory grief and then profound loss once death occurs.

Workers often mentioned going beyond their formal job descriptions to provide needed care to clients and peers at the end of life, largely because of the considerable lack of accessible services and supports for structurally vulnerable populations, but also the close, familial relationship they developed and moral responsibility they felt to alleviate suffering and provide needed care. Yet, despite taking on these additional “family” caregiver roles, many described how they were treated differently than families of origin by the health, legal, and social care systems. Not only did this lack of recognition create frustrating challenges logistically in the provision of end-of-life care (e.g. limited access to health information, legal wills, etc.), but it also amplified feelings of injustice, which had profound impacts on the grieving process.

### “Death surrounds me all the time”: Unveiling the complex layers of compounded grief

The grief and distress experienced by workers in this study was highly complex and layered. Participants explained how they were grieving not only for one particular person who was dying or had died, but for the substantial number of deaths that have occurred in their community over years. One secondary participant described how the street community has experienced “*profound loss,”* exclaiming: *“I’ve had 14 friends die in my arms.”* This cumulative grief is due in part to the ongoing drug poisoning crisis that continues to plague our region, but also the negative health outcomes and increased mortality the people they support experience due to their social positioning and deprivation in relation to social determinants of health. One primary participant explained, “*there is so much death happening, there is grief from* 20 *years ago. . . and then there is ‘now’ grief*,” highlighting how the rapid succession of deaths occurring in their community over time makes coping an overwhelming task. This participant exclaimed, “*somedays, I just want to. . .. ya know. . . cry. . .stand out in the parking lot at the shelter and just scream!.”*

Amplifying their cumulative grief is the additional layer relating to the structural violence workers must bear witness to on a daily basis. One primary participant described during an interview how her palliative homeless client was discharged from the hospital with no place to go, “*And then they die and everybody goes, ‘Oh my God, I wish we could’ve done something’ Well, the front-line workers roll up in a ball and just sob.”* Participants shared multiple traumatic accounts of their dying clients and peers falling through the cracks of the systems, receiving little to no support, suffering until the moment of their death, to after death witnessing bodies and belongings being treated in undignified ways. Furthermore, grief was found to be intertwined with feelings of guilt regarding whether they “*did enough*” for people they were supporting at the end of life as they tried to make up for a *“lifetime of suffering*.”

### “There is this ‘numbness’ for a lot of us folks”: Fear of opening the floodgates with no space or time to grieve

Participants commonly described the difficulties of navigating grief and distress as both a professional provider and “de facto” family member. Boundaries between work and life were described as incredibly nebulous, as this primary participant explained: “*I’m paid to provide a service, but that service is so loosely defined that you become really family.”* Workers felt emotionally conflicted over professional expectations, which encourages the development of close and caring client relationships, but to continue on with work “as usual” if a client dies. The emotional labor required to align their complex feelings/grief with professional expectations was described as onerous. Rather than providing grief/bereavement support, the workplace discourse was described by some participants as asserting blame onto workers for not maintaining healthy emotional boundaries. This participant explained, *“the messages I received were if you have feelings about clients, you have bad boundaries. If you have emotions then you are doing something wrong”*. Participants shared their disagreement surrounding the typical “boundary” discourse in their workplaces and how they had never received training on tools or guiding principles that acknowledges, and facilitates the processing of, these emotions in the context of providing care for dying clients and peers.

Workers commonly stated that despite being “*surrounded by death,”* recognition of their critical role in providing care for people at the end of life remains invisible and under-valued, resulting in few meaningful supports, including available work space or time to process grief. Consequently, participants described emotionally detaching as a coping strategy, which involved *“bottling it all up”*, *“feeling numb,”* and a fear of *“opening the floodgate. . . people don’t want to talk about it ‘cause it just spirals.”* The action team meetings provided space for participants to express emotions indicative of grief as well as an opportunity for participants to engage in meaningful actions to improve end-of-life conditions for their clients and peers. Participants reported how gaining greater knowledge of integrating a palliative approach to care in their everyday work (e.g. identifying people who could benefit, knowing what to expect, planning for future, getting needed supports) enabled them to access material supports in-line with their clients and peers’ wishes, diminishing their grief and distress.

## Discussion

Our analysis reveals how the relationships workers have with the people they support, due to the unique context of their work, results in them grieving as “de facto” family members, not just providers. Their grief is also complex, cumulative, and layered, reaching from the broad effects of structural inequities and violence that shapes the ways people live and die, to individual experiences of particular peoples’ deaths. Lastly, findings expose the difficulties workers experience in navigating their grief as both professionals and “de facto” family members and points to the need for greater recognition of their role as end-of-life caregivers and more tailored workplace supports to diminish distress and facilitate their grieving process.

Through a critical lens, these findings contribute greater understanding on the inequitable distribution of grief in our society by demonstrating how structurally vulnerable populations, and those who work tirelessly to provide their care through life and death, suffer from a disproportionate level of grief. With each new death, each example of injustice, grief is revived, compounded, and amplified, triggering ripples of anger, distress, and despair throughout the workers and street community. Ultimately, our findings exemplify how grief is not simply an individual/personal experience, but also an equity one,^1,2,8^ exposing how populations impacted by structural inequities and extreme social disadvantage are also denied the right to legitimize their grief experiences through acknowledging collective losses.^1^ The waves of death, loss, and grief that flow through these communities are the result of inequitable systems of power and failures of governance.^7,9,16^

### Implications for palliative care

Support for grief and bereavement are core components of palliative care provision.^31,32^ While it is often assumed that, in the community, palliative care takes place within traditionally defined “homes” where families of origin and/or friends become family caregivers, it is important to recognize that this care also occurs in subsidized housing facilities, shelters, food banks, and community outreach centers, among others, where community service workers are filling the gaps in formal services and in some cases, assuming palliative family caregiving roles.^3^ Thus, as “defacto family,” they deserve access to the same level of support for grief and bereavement as other family caregivers, but these supports must be tailored to the unique context in which they live and work. Overall, our research puts forth a call to formally recognize and value community service workers in these palliative caregiving roles and for organizations/employers to work toward ensuring their employees have access to the supports they need to mitigate the risk of complex grief, grief fatigue, and burnout.^20,22,33,34^

Collective grief is experienced in these communities from the ongoing structural inequities and violence occurring.^20^ As such, a collective and material response is required, which includes witnessing, acknowledging, and valuing their grief and the lives they are grieving. For example, rather than pathologizing grief as individual experiences^35^ reflective of poor professional boundaries,^3,22^ it should be recognized and valued as a likely outcome from quality care provision.^33^ Interventions and supports may need to take a more “side-door” approach, recognizing the complexity of engaging in emotional debriefing sessions, and focusing instead on facilitating collective care/grieving, community wellness, community advocacy projects and public mourning that are empowering, therapeutic, and reinforce resilience and the value of “caring” for workers. Organizational shifts toward an equity-informed palliative approach to care may help provide needed support to workers in their current role of providing palliative care to the most vulnerable populations in our society.

### Strengths and weaknesses

This study deepens understanding on the complex experiences of grief that community service workers endure in the context of providing care to structurally vulnerable people at the end of life. It not only casts a spotlight upon this invisible emotional labor, but offers suggestions on promising directions to enhance supports for these workers, which are more accessible, meaningful, and equitable, facilitating their capacity to continue providing this needed care. While our findings provide direction on the unique support requirements of these workers, this analysis was drawn from data pertaining to a larger participatory action research study where grief and distress were not the focus. Further research examining the complex and nuanced intersections of workers’ grief, care for structurally vulnerable populations, and palliative care may thus be warranted. Lastly, a need exists for more research to examine inequitable distributions of grief and the various ways that social locations, structures, and systems coalesce to shape differing workers’ experiences.
